# Digital Screening and Automated Resource Identification System to Address COVID-19–Related Behavioral Health Disparities: Feasibility Study

**DOI:** 10.2196/38162

**Published:** 2022-06-22

**Authors:** Colleen Stiles-Shields, Kathryn R Batts, Karen M Reyes, Joseph Archer, Sharad Crosby, Janel M Draxler, Nia Lennan, Philip Held

**Affiliations:** 1 Section of Community Behavioral Health Department of Psychiatry and Behavioral Sciences Rush University Medical Center Chicago, IL United States; 2 Community Health Research Division RTI International Research Triangle Park, NC United States; 3 University of Wisconsin School of Medicine and Public Health Madison, WI United States; 4 Institute for Translational Medicine University of Chicago Chicago, IL United States

**Keywords:** digital mental health, underserved, health disparities, COVID-19, screening, referral, mental health, digital health, feasibility study, mobile app, mHealth, mobile health, emotional need, digital health tool, health resource, health care cost

## Abstract

**Background:**

Digital mental health (DMH) tools use technology (eg, websites and mobile apps) to conveniently deliver mental health resources to users in real time, reducing access barriers. Underserved communities facing health care provider shortages and limited mental health resources may benefit from DMH tools, as these tools can help improve access to resources.

**Objective:**

This study described the development and feasibility evaluation of the Emotional Needs Evaluation and Resource Guide for You (ENERGY) System, a DMH tool to meet the mental health and resource needs of youth and their families developed in the context of the COVID-19 pandemic. The ENERGY System offers a brief assessment of resource needs; problem-solving capabilities; and symptoms of depression, anxiety, trauma, and alcohol and substance use followed by automated, personalized feedback based on the participant’s responses.

**Methods:**

Individuals aged ≥15 years were recruited through community partners, community events, targeted electronic health record messages, and social media. Participants completed screening questions to establish eligibility, entered demographic information, and completed the ENERGY System assessment. Based on the participant’s responses, the ENERGY System immediately delivered digital resources tailored to their identified areas of need (eg, relaxation). A subset of participants also voluntarily completed the following: COVID-19 Exposure and Family Impact Survey (CEFIS) or COVID-19 Exposure and Family Impact Survey Adolescent and Young Adult Version (CEFIS-AYA); resource needs assessment; and feedback on their experience using the ENERGY System. If resource needs (eg, housing and food insecurity) were endorsed, lists of local resources were provided.

**Results:**

A total of 212 individuals accessed the ENERGY System link, of which 96 (45.3%) completed the screening tool and 86 (40.6%) received resources. Participant responses on the mental health screening questions triggered on average 2.04 (SD 1.94) intervention domains. Behavioral Activation/Increasing Activities was the most frequently launched intervention domain (56%, 54/96), and domains related to alcohol or substance use were the least frequent (4%, 4/96). The most frequently requested support areas were finances (33%, 32/96), transportation (26%, 25/96), and food (24%, 23/96). The CEFIS and CEFIS-AYA indicated higher than average impacts from the pandemic (ie, average scores >2.5). Participants were satisfied with the ENERGY System overall (65%, 39/60) as well as the length of time it took to answer the questions (90%, 54/60), which they found easy to answer (87%, 52/60).

**Conclusions:**

This study provided initial support for the feasibility of the ENERGY System, a DMH tool capable of screening for resource and mental health needs and providing automated, personalized, and free resources and techniques to meet the identified needs. Future studies should seek direct feedback from community members to further improve the ENERGY System and its dissemination to encourage use.

## Introduction

### Background

Prior to the COVID-19 pandemic, nearly half of all adolescents and a quarter of all adults in the United States met the criteria for at least one mental health disorder [1,2]. The effects of the COVID-19 pandemic have exacerbated mental health concerns, particularly for adolescents and young adults [3-5]. Further, structural barriers that existed prior to the COVID-19 pandemic primed specific communities to disproportionately experience negative effects from the pandemic [6,7]. However, the majority of these individuals have not received mental health care [8,9]. Mental health stigma, time limitations, and the identification of mental health problems were among the primary barriers to accessing care [10-20]. Additionally, the demand for behavioral health care frequently exceeds clinic capacity, particularly in rural areas [21]. Given the lack of accessible mental health services and the increased need for such resources, it is imperative to identify novel means for providing behavioral health assessment and care, particularly to members of vulnerable communities.

Digital mental health (DMH) tools are one way to enhance the accessibility of evidence-based interventions and resources [22]. DMH refers to the use of technology platforms, such as websites (ie, eHealth), mobile apps (ie, mobile health), and electronic devices (eg, Fitbit and Apple Watch), to deliver behavioral health assessments and interventions. The use of DMH has expanded rapidly during the COVID-19 pandemic [23], and there are multiple reasons it represents a viable means to provide continued behavioral health resources, particularly for communities disproportionately facing access barriers. First, DMH tools can assess and offer resources for use in real time and real-world environments without requiring individuals to travel to mental health care settings and allow for asynchronous, socially distanced care [24]. Further, DMH can also link to local or web-based resources for basic needs (eg, housing support); facilitating access to resources that support basic physiological needs is consistent with tools aimed at supporting higher level needs, such as mental health [25]. Second, mobile device access is ubiquitous [26], and the use of DMH therefore increases the likelihood of engaging individuals through technology that they are already using (eg, smartphones and tablets). Finally, youths are more likely to seek and access emotional support through technology compared to in-person care [26,27], and the pandemic has increased technology use and ownership across all ages, including older adults [28]. This makes DMH a delivery mechanism that harnesses an environment in which those at the highest risk for pandemic-related mental health concerns are more likely to feel comfortable. Importantly, DMH tools developed prior to and during the COVID-19 pandemic have the potential to extend in utility beyond the current pandemic to potential future crises [29].

The digital marketplace for DMH tools can be overwhelming with app stores featuring over 10,000 publicly available mobile health apps. The majority of available DMH tools are either not based on evidence-based interventions or have not been rigorously evaluated [28]. In addition to the overwhelming amount of available DMH tools, most require users to have already identified the problems for which they are seeking help, which can be difficult for some [30]. Thus, to make DMH more accessible to vulnerable populations, potential users must be able to identify (1) what problems they are having (eg, anxiety and depression, etc) and (2) which tools are appropriate and efficacious for their needs. Moreover, it is important that interventions be transparent and specifically targeted toward vulnerable groups and adapted to their needs.

### Objective

To address these challenges, we developed a DMH tool, the Emotional Needs Evaluation and Resource Guide for You (ENERGY) System, to briefly assess mental health and other resource needs and provide automated, personalized feedback based on the identified needs. The objective of this study was to develop the tool and assess its feasibility (ie, engagement and satisfaction) to guide future developments. The ENERGY System was originally designed in the context of the COVID-19 pandemic for adolescents and young adults (AYAs) and their families residing in the West and South Side communities of Chicago but was ultimately deployed throughout the city and surrounding neighborhoods of Chicago, Illinois. These communities have faced stark mental and behavioral health disparities, both prior to and during the pandemic [7,31-33].

## Methods

### Participants

Participants were recruited from the Chicagoland area (ie, the city of Chicago, Illinois, and its surrounding suburbs and towns). Recruitment was conducted via (1) the distribution of flyers that contained the study link and QR code to the Rush University Education and Career Hub and local community partners and at community events (eg, Easter egg hunt and vaccine clinics); (2) the posting of flyers and short videos on social media platforms (eg, departmental Twitter account); and (3) targeted messaging to electronic health records (eg, the Epic MyChart app) of patients receiving services from 3 school-based health centers located in Chicago’s West Side communities. The initial deployment of the ENERGY System in March 2021 limited participation to people aged 15-25 years or caregivers of children aged <18 years. However, due to community needs and recognition that families with complex needs extend beyond these subgroups, the inclusion criteria were expanded at the end of May 2021 to include anyone aged ≥15 years.

### Ethics Approval

The study procedures were approved by the Rush University Medical Center Institutional Review Board (20092408).

### Enrollment Procedure

Waivers of the documentation of informed consent and parental permission as well as an alteration of consent were obtained. Interested individuals accessed the system via a REDCap [34] link provided on recruitment materials (eg, physical flyers, social media posts, and email blurbs); the REDCap link opened a page on the participant’s personal device’s web browser. REDCap was used to increase accessibility, as users would not be required to download anything to their device to use the ENERGY System. After clicking the link, participants were asked to enter their age and whether they are a parent of a child aged <18 years. Eligible persons were directed to a brief form listing all the required elements of consent. Participants were asked to click “I agree” to continue to the ENERGY System. Those who did not agree to the elements of consent were exited from the system.

### ENERGY System

#### System Overview

The ENERGY System is a brief, DMH-screening, and automated resource identification system. The ENERGY System was developed to provide individuals with an automated tool that can identify various emotional and mental health concerns and provide an automated list of appropriate self-help and other resources to address these identified concerns. Automating the screening and resource identification process was intended to improve the accessibility of such services, which are usually performed by mental health or social service providers and thus have limited scalability. Although the ENERGY System was administered via REDCap [34] for this study, it can also be transported to other platforms.

#### ENERGY System Development

The ENERGY System was designed to minimize the burden related to the assessment of current needs and concerns and maximize the feasibility. To achieve this, the mental health screening portion of the ENERGY System combines 16 items drawn from existing, validated scales assessing problem-solving issues, depression and anxiety symptoms, as well as alcohol and substance use into a single survey. The items used in the ENERGY System were derived from prior research on the development of brief assessments of each of these constructs. Specifically, the items used in the ENERGY System were drawn from the 7-item Generalized Anxiety Disorder Assessment (GAD-7) [35], 5-item PTSD Checklist for DSM-5 (PCL-5) [36], 9-item Patient Health Questionnaire (PHQ-9) [37], 3-item Alcohol Use Disorder Identification Test-Consumption (AUDIT-C) [38], and Drug Abuse Screening Test (DAST-10) [39]. In a separate research study (Christ et al, unpublished data, 2022), our team used Item Response Theory on several of these short forms and the full scales from which they were derived to identify specific items that best represent the underlying latent constructs. For each brief scale derived based on Item Response Theory, the associations with the respective full scales as well as related constructs were assessed. Furthermore, our team evaluated the predictive validity of these brief scales by examining how changes in the brief scales predicted changes in the related constructs over the course of treatment and how this compared to the full scales (Christ et al, unpublished data, 2022). For the ENERGY System, we only retained items/brief scales that explained the underlying constructs and were able to adequately capture changes in these constructs following an intervention. This step was important to ensure that the ENERGY System would be able to accurately reflect changes in the underlying constructs when administered repeatedly. The resulting 16-item ENERGY System uses 2 items from the PCL-5, 4 items from the PHQ-9, 3 items from the GAD-7, 3 items from the AUDIT-C, 3 items from the DAST-10, and 1 author-developed item asking about participants’ ability to handle problems.

The ENERGY System screening items were then mapped onto 6 different intervention domains. Anxiety-related symptoms were mapped to Relaxation strategies. Depressive symptoms that were primarily associated with the lack of activity were mapped to Behavioral Activation strategies. Anxiety and depressive symptoms that were primarily associated with thinking processes were mapped to Cognitive Restructuring strategies. Problem-solving was mapped to Specific, Measurable, Achievable, Relevant, and Time-bound goals and other Problem-solving strategies. Alcohol and substance use were mapped to Alcohol and Substance Use Management and Harm Reduction strategies. These domains were created to identify specific intervention targets that could be more easily matched to specific evidence-based cognitive behavioral skills. For example, although anxiety is a single construct, different interventions may be more effective depending on the symptom presentation. An individual with anxiety symptoms of feeling on edge or having difficulty relaxing may benefit more from exercises focused on activating the parasympathetic nervous system (eg, diaphragmatic breathing, progressive muscle relaxation, and mindfulness), whereas an individual who has difficulty managing their worried thoughts may benefit from cognitive restructuring. Expert-reviewed resources, including MindTools [40] and PsyberGuide [41], were used to create a repository of DMH tools that have been shown to be beneficial for depression, anxiety, alcohol use, substance use, and problem-solving within personalized resources (eg, COVID Coach App [42]).

In addition to assessing specific emotional and mental health concerns, a goal of the ENERGY System was to capture resource needs and provide immediate, relevant information about readily accessible resources specific to the location in which the ENERGY System was first tested (ie, the West and South Side communities of Chicago). Namely, participants were directed to indicate whether they needed assistance with any of the following domains: housing, finances, parenting, childcare, food, transportation, medical care, pet care, elderly care, and any other miscellaneous areas of concern. Participants clicked a box next to any domain for which they wanted to receive assistance. These domains were selected because deficits in these areas have been associated with stress and negative impacts on individuals and their families in the short and long term [43]. Local community resources were identified through various methods. A web-based search for local Chicago resources (eg, lists of local food banks and rent/mortgage assistance programs) was conducted, and recommendations for COVID-19 testing sites and community safety hotlines were included to meet the needs of Chicago residents. Additionally, the team incorporated pertinent resources from a pre-existing list of community resources created by a team member for use during clinical care. Community resources were also identified through organizational LISTSERV software applications, colleagues, and a list of resources sent by a local congressman to his constituents. All community resources were checked for accuracy to ensure that they were still available as advertised prior to publishing them into recommendations for study participants.

#### ENERGY System Mental Health and Resource Feedback

Immediately upon the completion of the screening questions, the ENERGY System automatically scored the assessments to identify the domains that could benefit from intervention. Positive endorsement of an intervention domain was defined as a severity rating of at least 50% of the total possible score for the domain. For each positive domain, the ENERGY System automatically provided the individual with personalized feedback (eg, “Based on your responses, you may benefit from learning some relaxation strategies”) and community-based and digital resources tailored to participant responses. Resources were presented in a person-focused rather than clinical manner (eg, using terms such as “sadness” rather than “depression”) to reduce stigma.

The emotional and mental health feedback involved specific cognitive behavioral techniques that have been identified in prior research to be particularly effective for the respective concerns. For example, individuals who screened positive for sadness were introduced to behavioral activation techniques (referred to as “Increasing Activities”). Those who screened positive for symptoms of anxiety were provided tips and resources around progressive muscle relaxation, self-soothing, and meditation (referred to as “Relaxation”). All techniques were framed in a self-guided format by cognitive-behaviorally trained psychologists and described so that they could be easily followed by users without requiring coaching from a professional. In addition to detailed instructions, links to publicly available resources explaining these techniques (eg, YouTube) were provided to present individuals with added options for learning the different skills.

Feedback about other available resources was presented in a similar manner. Requested resources were presented as brief excerpts detailing the types of services provided and linking individuals to the agencies’ websites where more details could be found. Each resource opened to a new tab so that individuals could keep their list of recommended resources open in their browser for as long as necessary. Individuals also had the option to enter their email address to have their list automatically emailed to them. The resources feedback page also contained the link to the ENERGY System for the individual to visit again at any time or to share with others.

### Measures

#### Demographic Characteristics

Participants were asked to provide demographic characteristics, including age, gender, ethnicity, race, sexual orientation, the highest level of education, current student/employment status, family’s total annual income before taxes, zip code, and parental status.

#### ENERGY System

As described above, the ENERGY System assessment consisted of 16 items drawn from previously validated symptom scales. The reliabilities of the items in each of the intervention domains were adequate to good (Relaxation: Cronbach α=.77; Behavioral Activation/Increasing Activities: Cronbach α=.83; and Cognitive Restructuring [Low Mood]: Cronbach α=.86). Cronbach α was not calculated for Problem-solving as this was assessed via a single item. Cronbach α’s were also not calculated for Alcohol and Substance Use Management as items on the underlying scales were only triggered after the initial question about any alcohol or substance use was positively endorsed. Thus, participants had a widely varying number of responses to these items.

#### ENERGY System Satisfaction

Immediately after reviewing their recommended resources, participants were given the opportunity to provide overall feedback about the ENERGY System. Elicited feedback involved overall satisfaction and satisfaction with the ease of answering the questions, amount of time it took to complete, and recommended resources. Participants were also asked how likely they were to use the resources in the future and recommend the ENERGY System to others. Finally, participants were asked whether they would be interested in receiving professional support for resources or emotional needs, such as via connection to a therapist or primary care provider, if this were to be offered in the future.

#### COVID-19 Exposure and Family Impact

The COVID-19 Exposure and Family Impact Survey (CEFIS) [44] or the COVID-19 Exposure and Family Impact Survey Adolescent and Young Adult Version (CEFIS-AYA) [45] were administered, depending on age (CEFIS for all adults aged ≥30 years and CEFIS-AYA for AYAs aged 15-29 years). The CEFIS and CEFIS-AYA assess the impact of the COVID-19 pandemic on adolescents, young adults, and families. The CEFIS yields (1) an Exposure score (scores range from 0-25, with higher scores indicating greater exposure); (2) an Impact score (scores averaged from a 4-point Likert scale with scores >2.5 considered positively valenced, meaning more impact, and scores <2.5 considered negatively valenced, meaning less impact); (3) a Distress score (scores range from 1-10, with higher scores indicating greater distress); and (4) an open-ended question to promote the sharing of details not covered. The CEFIS-AYA differs from the CEFIS by having 3 additional Exposure items, 6 additional Impact items, and a single item for the Distress scale to reflect the experience of AYAs compared to that of a family. The CEFIS demonstrated acceptable reliability for the current sample (αs>.73). However, due to the possibility that adult respondents completed the CEFIS without having children, the CEFIS Distress was reported at the item level (personal and child, separately) as opposed to the scale level (personal and child combined). The CEFIS-AYA demonstrated acceptable reliability across subscales (αs>.69).

### Data Analysis

Descriptive analyses were used to (1) characterize the sample in terms of demographic characteristics and reported resource needs; (2) determine use rates; (3) identify the total provided mental health resources; and (4) assess user satisfaction with the ENERGY System.

## Results

### Participants

Table 1 depicts the demographic characteristics of the sample. Of the 110 participants who provided demographic data, they were primarily AYAs (68.2%, n=75), cisgender female (81.3%, n=91), heterosexual or straight (67.6%, n=75), non-Hispanic/Latinx (67.3%, n=74), and Black or African American (40.9%, n=45) or White (40%, n=44). A total of 103 (93.6%) participants provided their zip codes. Of these 103 participants, the majority (95.1%, n=98) lived in Illinois, with 80 (77.7%) residing within the city limits of Chicago. The remaining 5 (4.9%) were from California, Indiana, New York, Texas, and Wisconsin.

**Table 1:** Participant characteristics.

Characteristic		Adolescents and young adults^a^ (n=75)	Adults^a^ (n=35)	All participants (N=110)
Age (years), mean (SD; range)		18.95 (3.18; 15-25)	43.89 (13.94; 26-71)	26.88 (14.27; 15-71)
**Gender, n (%)**				
	Cisgender female	68 (90.7)	22 (62.9)	90 (81.8)
	Cisgender male	4 (5.3)	11 (31.4)	15 (13.6)
	Transgender female	0 (0)	1 (2.9)	1 (0.9)
	Transgender male	1 (1.3)	1 (2.9)	2 (1.8)
	Nonbinary	1 (1.3)	0 (0)	1 (0.9)
	Prefer not to answer	1 (1.3)	0 (0)	1 (0.9)
**Ethnicity, n (%)**				
	Hispanic/Latinx	28 (37.3)	7 (20)	35 (31.8)
	Non-Hispanic/Latinx	47 (62.7)	27 (77.1)	74 (67.3)
	Prefer not to answer	0 (0)	1 (2.9)	1 (0.9)
**Race^b^, n (%)**				
	American Indian or Alaskan Native	1 (1.3)	1 (2.9)	2 (1.8)
	Asian	5 (6.7)	2 (5.7)	7 (6.4)
	Black or African American	38 (50.7)	7 (20)	45 (40.9)
	White	20 (26.7)	24 (68.6)	44 (40)
	Other	5 (6.7)	1 (2.9)	6 (5.5)
	Prefer not to answer	10 (13.3)	1 (2.9)	11 (10)
**Sexual orientation, n (%)**				
	Heterosexual or straight	51 (68)	24 (68.6)	75 (68.2)
	Gay or lesbian	3 (4)	5 (14.3)	8 (7.3)
	Bisexual or pansexual	18 (24)	4 (11.4)	22 (20)
	Asexual	0 (0)	1 (2.9)	1 (0.9)
	Other	1 (1.3)	0 (0)	1 (0.9)
	Prefer not to answer	2 (2.7)	1 (2.9)	3 (2.7)
**Highest level of education, n (%)**				
	Finished grade school	5 (6.7)	0 (0)	5 (4.5)
	Some high school	29 (38.7)	0 (0)	29 (26.4)
	Finished high school	9 (12)	0 (0)	9 (8.2)
	Business or technical school	0 (0)	1 (2.9)	1 (0.9)
	Some college	21 (28)	5 (14.3)	26 (23.6)
	Finished college	8 (10.7)	8 (22.9)	16 (14.5)
	Some graduate or professional school	1 (1.3)	6 (17.1)	7 (6.4)
	Finished graduate or professional school	2 (2.7)	15 (42.9)	17 (15.5)

^a^Adolescents and young adults were aged 15-25 years and adults were aged ≥26 years.

^b^The total number of reported racial identities is 115 due to participants identifying with more than one racial category.

### ENERGY System Use Data

**Figure 1:**
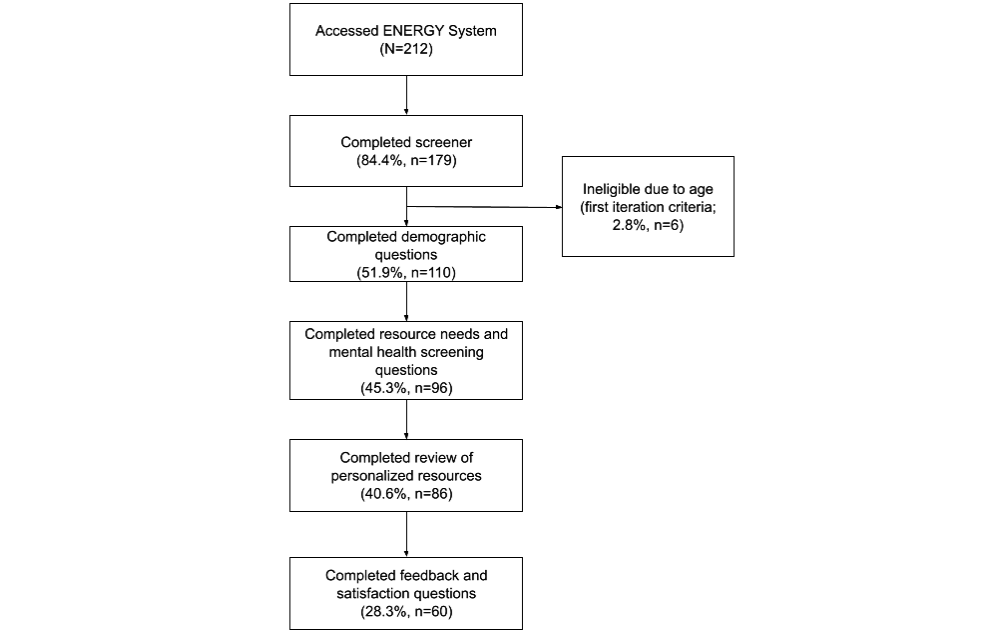
Flow chart of participants through ENERGY System use. ENERGY: Emotional Needs Evaluation and Resource Guide for You.

Between August 2020 and December 2021, the ENERGY System link was accessed 212 times. Attrition occurred with each advancement of the system, with 84.4% (n=179) completing the screening questions, 51.9% (n=110) completing the demographic questions, 45.3% (n=96) completing the resource needs and mental health screening, 40.6% (n=86) completing the review of their personalized resources, and 28.3% (n=60) providing feedback about the ENERGY System. On average, participants completed their interactions with the system in 12 (SD 8.33) minutes (6 outliers were removed from the average calculation as it was believed that they completed their interactions across multiple visits [ie, >1 hour], with a range from 1 hour and 20 minutes to 23 hours and 29 minutes). Figure 1 depicts the flow chart for the ENERGY System interactions.

### Mental Health Intervention Domains

A total of 96 participants (45.3%, N=212) completed the mental health screening questions. Participant responses on the mental health screening questions triggered on average 2.04 (SD 1.94) intervention domains. Of the 96 responses, Behavioral Activation/Increasing Activities was the most launched intervention domain (56%, n=54), followed by Relaxation (44%, n=42), Problem-solving (41%, n=39), and Cognitive Restructuring (Low Mood; 32%, n=31). Alcohol Use and Substance Use were the least frequently launched intervention domains, launching only 4 (4%) times each.

### Resource Needs

A total of 96 participants (45.3%, N=212) completed resource needs questions, with half (50%, n=48) responding in a way that indicated that they did not need assistance with any resources. As such, the average number of resource categories provided to participants was 0.69 (SD1.44; range 0-7). Of the 96 responses, the endorsed resource needs included financial support (33%, n=32), transportation (26%, n=25), food (24%, n=23), housing (17%, n=16), medical care (14%, n=13), parenting (12%, n=12), pet care (11%, n=11), childcare (10%, n=10), and resources for caretakers of older adults (5%, n=5).

### COVID-19 Exposure and Impact

A total of 64 AYA participants and 24 adult participants completed some portion of the CEFIS-AYA and CEFIS, respectively. To limit participant burden, Exposure subscale items were offered optionally and completed by 16 (25%, N=64) AYAs and 17 (71%, N=24) adults. The AYAs and adults had an average Exposure rating of 13.13 (SD 4.00; range 6-20) and 8.65 (SD 3.02; range 3-16), respectively. Both the AYA and adult Impact subscale scores were positively valenced (more impact), with AYAs having an average Impact score of 2.82 (SD 0.65; range 1.21-4.00) and adults having an average Impact score of 3.00 (SD 0.52; range 2.10-3.78). AYAs endorsed an average personal Distress rating of 5.78 (SD 2.44; range 1-10). Adults reported their personal Distress on average as 6.46 (SD 2.41; range 2-10) and their children’s Distress was rated on average as 4.64 (SD 3.01; range 1-10).

### ENERGY System Satisfaction

A total of 60 (28.3%, N=212) participants provided feedback about the ENERGY System following use. Of the 60 participants, the majority agreed or strongly agreed that they were satisfied with the system overall (65%, n=39), ease of answering questions (87%, n=52), and time it took to answer questions (90%, n=54). The majority (75%, n=45) also agreed or strongly agreed that the ENERGY System asked about all of their needs. Finally, the majority (78%, n=47) somewhat agreed, agreed, or strongly agreed that they were likely to use the system’s recommended resources.

## Discussion

### Principal Findings

This study aimed to develop and assess the feasibility of a DMH tool that (1) screened for resource and mental health needs and (2) provided automated, personalized, and free resources and techniques to meet the identified needs. Dissemination efforts focused on institutional and community partner collaborations to focus on use by residents of communities facing disproportionate behavioral health disparities. Over 200 community members, largely within the Chicagoland area, demonstrated initial interest in the ENERGY System. However, attrition occurred with each progressing stage of the system (ie, screener, demographic questions, resource needs and mental health screening assessment, review of personalized resources, and system feedback). Half of all individuals who completed the screening questions endorsed not having resource needs. On average, individuals received resources for 2 mental health symptoms and lists based on their responses. As is common for subjective evaluations of DMH, even in the face of relatively low real-world engagement [46], users who provided feedback were primarily satisfied with the system.

The current sample endorsed higher than average impacts from the COVID-19 pandemic, with AYAs reporting higher exposure to pandemic experiences than their adult counterparts [44]. These experiences have occurred within the context of historical and current behavioral health disparities and broader community hardships [32]. As such, the COVID-19 pandemic has exacerbated the already existent need for scalable, accessible, and affordable DMH, particularly for vulnerable communities [47]. Although multiple studies of pandemic-specific DMH interventions have occurred [48], the ENERGY System is unique in its intended use (ie, single or repeated use) and potential for extended utility beyond the pandemic (eg, compliant with but not centered upon social distancing practices). However, to be useful for communities moving forward, multiple limitations will need to be addressed.

### Accessibility

The ENERGY System was intended to serve those facing the greatest hardships and disparities from the pandemic. However, the majority of sample members denied requiring resource needs, and about 40% of the adults who used the system had a graduate or professional degree. These findings likely indicate that our recruitment efforts did not reach many of the residents requiring this additional support. Further, slightly over 200 initial clicks to the system suggest that the deployment had a low reach (eg, Chicago's Lower West Side community has a population of about 12,000 people per square mile) [49]. Therefore, increasing accessibility is an important factor for future deployments of the ENERGY System and similar DMH tools. The ENERGY System was designed for an immediate need and in the context of social distancing mandates. However, best practices would include the incorporation of human-centered design [50-52] and community-based participatory research methodologies [53] to ensure that likely end users are active collaborators—those with agency—throughout the design and deployment phases. Doing so better ensures that DMH is accessible to and appropriate for the intended users [22,54]. For example, nearly one-third of the sample identified as Hispanic or Latinx, but the tool was only available in the English language. The use of human-centered design practices with community members would provide ongoing assessment of likely end users’ native languages and language preferences for DMH, promoting the likelihood of increasing accessibility to nonnative English speakers.

### Engagement

Less than half of the sample members completed the resource needs and mental health screening questions. The inclusion of additional questions for the purposes of research (eg, CEFIS and satisfaction questions) likely contributed to some of the attrition that occurred with the ENERGY System in this study. We note that engagement with DMH in real-world settings is generally low [55], with top barriers including (1) stigma, (2) problem recognition, and (3) knowledge of treatment options [10-12]. The ENERGY System is designed to address these concerns (ie, anonymous participation on a personal device to address stigma; feedback about mental health symptoms to address problem recognition; and free resources provided to address knowledge of treatment options). However, engagement with the system was low. These findings again support the need to involve representative potential users throughout the design process. It is unsurprising that community members systemically experiencing disproportionate disparities may not engage well with DMH in real-world settings, as DMH has historically been developed without their input regarding their lived experiences and needs [56]. A focus on engagement strategies with systemically excluded and marginalized community members must also be a focus of future design and research.

### Limitations

This study should be considered in light of its specific limitations. First, social distancing mandates limited recruitment efforts. Namely, typical recruitment methods of community partners often involve in-person communication (even in the case of sharing a website link or QR code), which was limited due to the pandemic. A large portion of flier dissemination occurred through mass emails; this methodology likely resulted in study materials being overshadowed by other web-based obligations [57,58]. Second, remote learning or e-learning limited the ability of recruitment partners in school settings to engage and explain the study to younger potential participants. Third, the ENERGY System was only provided in the English language. Roughly 16% of Chicago residents do not speak English as a primary language, with Spanish being the most frequently spoken language among this group [59]. This barrier left several Chicago families unable to complete the screener. Future resource tools similar to the ENERGY System should create multiple translations, based on formative assessments of preferred language, for optimal use among diverse communities. Finally, the ENERGY System was disseminated through the Rush University Medical Center clinics and community partnerships. Although some users may trust DMH when overseen by a university setting due to institutional review board oversight, others are less inclined to trust university and larger health systems–sponsored DMH due to historical injustices [60]. Future research should collaborate with community members to assess how the role of academic institution involvement may promote or hinder DMH engagement.

### Conclusions

This study provided initial support for the feasibility of the ENERGY System, a DMH tool capable of screening for resource and mental health needs and providing automated, personalized, and free resources and techniques to meet the identified needs. Future research should involve collaboration with community members and collect more detailed formative and summative data from representative end users of the ENERGY System. As such, better attempts to meet the needs of users with intersectional identities and varying mental health and resource needs may result in improved engagement [46]. Additionally, larger scale research is needed to determine the feasibility of the ENERGY System beyond the areas targeted in this study. Moreover, longitudinal studies are needed to determine whether individuals use the resources and mental health skills they were provided and whether the use of skills and resources improves their reported symptoms over time.

## Abbreviations

AUDIT-C: 3-item Alcohol Use Disorder Identification Test-Consumption
AYA: adolescent and young adult
CEFIS: COVID-19 Exposure and Family Impact Survey
CEFIS-AYA: COVID-19 Exposure and Family Impact Survey Adolescent and Young Adult Version
DAST-10: Drug Abuse Screening Test
DMH: digital mental health
ENERGY: Emotional Needs Evaluation and Resource Guide for You
GAD-7: 7-item Generalized Anxiety Disorder Assessment
PCL-5: 5-item PTSD Checklist for DSM-5
PHQ-9: 9-item Patient Health Questionnaire
